# NAADP‐induced intracellular calcium ion is mediated by the TPCs (two‐pore channels) in hypoxia‐induced pulmonary arterial hypertension

**DOI:** 10.1111/jcmm.16783

**Published:** 2021-07-15

**Authors:** Wen Hu, Fei Zhao, Ling Chen, Jiamin Ni, Yongliang Jiang

**Affiliations:** ^1^ Respiratory Medicine Hunan Provincial People's Hospital Changsha China

**Keywords:** [Ca^2+^]*_i_*, pulmonary arterial hypertension (PAH), pulmonary arterial smooth muscle cells (PASMC), pulmonary artery endothelial cells (PAECs), two‐pore segment channel (TPC)

## Abstract

Pulmonary arterial hypertension (PAH) is a form of obstructive vascular disease. Chronic hypoxic exposure leads to excessive proliferation of pulmonary arterial smooth muscle cells and pulmonary arterial endothelial cells. This condition can potentially be aggravated by [Ca^2+^] *_i_* mobilization. In the present study, hypoxia exposure of rat's model was established. Two‐pore segment channels (TPCs) silencing was achieved in rats' models by injecting Lsh‐TPC1 or Lsh‐TPC2. The effects of TPC1/2 silencing on PAH were evaluated by H&E staining detecting pulmonary artery wall thickness and ELISA assay kit detecting NAADP concentrations in lung tissues. TPC1/2 silencing was achieved in PASMCs and PAECs, and cell proliferation was detected by MTT and BrdU incorporation assays. As the results shown, NAADP‐activated [Ca^2+^]*_i_* shows to be mediated via two‐pore segment channels (TPCs) in PASMCs, with TPC1 being the dominant subtype. NAADP generation and TPC1/2 mRNA and protein levels were elevated in the hypoxia‐induced rat PAH model; NAADP was positively correlated with TPC1 and TPC2 expression, respectively. In vivo, Lsh‐TPC1 or Lsh‐TPC2 infection significantly improved the mean pulmonary artery pressure and PAH morphology. In vitro, TPC1 silencing inhibited NAADP‐AM‐induced PASMC proliferation and [Ca^2+^]*_i_* in PASMCs, whereas TPC2 silencing had minor effects during this process; TPC2 silencing attenuated NAADP‐AM‐ induced [Ca^2+^]*_i_* and ECM in endothelial cells, whereas TPC1 silencing barely ensued any physiological changes. In conclusion, TPC1/2 might provide a unifying mechanism within pulmonary arterial hypertension, which can potentially be regarded as a therapeutic target.

## 1 INTRODUCTION

PAH (pulmonary arterial hypertension) is characterized as a progressive, chronic and crippling disease related to abnormally high blood pressure within pulmonary arteries. PAH is caused by morphological changes in the precapillary pulmonary vessels.^1^, ^2^, ^3^, ^4^ As a result, PAH is associated with morbidity and fatality in adults and children suffering from a range of cardiac and pulmonary disorders.^5^

An array of factors, including aberrant cell proliferation in the vascular wall, the loss of endothelial cell function and vascular remodelling accompanied with extracellular matrix (ECM) overdeposition in endothelial cells, can potentially contribute to PAH progression.^6^, ^7^, ^8^ Excessive proliferation in small pulmonary arteries, including PASMCs (pulmonary artery smooth muscle cells) and PAECs (pulmonary artery endothelial cells), endothelial colony‐forming cells (ECFCs) could potentially trigger vascular remodelling, which alters the pulmonary arteries structures along with its biochemical and functional phenotypes.^9^, ^10^ An in‐depth understanding of the mechanism of the regulation of PASMC and PAEC proliferation could potentially help vascular remodelling and therefore contribute towards PAH alleviation.

Cytoplasmic Ca^2+^ mobilization plays a crucial role in pulmonary vascular remodelling caused by chronic hypoxia.^11^, ^12^, ^13^ Ca^2+^ signalling is enhanced in patients with idiopathic PAH. The elevated concentration of cytosolic free calcium within PASMCs can be regarded as a main inducing factor of pulmonary vasoconstriction and trigger calcium‐sensitive signalling cascades through calmodulin elicit an increase in the proliferating ability of cells.^14^, ^15^ Therefore, elevated calcium signalling can induce sustained pulmonary vasoconstriction and trigger the thickening of pulmonary vascular walls by enhancing the ability of PASMCs to proliferate and migrate, subsequently promoting the pathogenesis of pulmonary arterial hypertension. Within PAEC, CCE (capacitative Ca^2+^ entry) via SOC (store‐operated Ca^2+^ channels) is considered as a critical mechanism for enhancing cytosolic free [Ca^2+^].^16^, ^17^, ^18^, ^19^ The Ca^2+^‐induced elevation in protein (AP)‐1 binding activity within PAEC is an essential process in AP‐1‐responsive gene up‐regulation, pulmonary vascular cell proliferation induction and the vascular remodelling of the lungs of hypoxia‐induced PAH patients.^20^

The increase in cytoplasmic calcium is controlled by calcium‐mobilizing messengers. For example, the application of NAADP (nicotinic acid adenine dinucleotide phosphate), a known calcium‐mobilizing molecule, to microsomes of aortic SMC (smooth muscle cells) induced calcium release.^21^, ^22^ Two‐pore channels (TPC) 2 have been reported to be one of the TPCs which could be triggered via NAADP to release calcium from endolysosomal organelles.^23^, ^24^, ^25^ Moccia et al found that NAADP‐induced TPC1‐mediated Ca^2+^ release can selectively be recruited to induce the Ca^2+^ response to specific cues in circulating ECFCs.^26^ The expression of TPC1/2 within PASMCs of rats has been proven in our previous study, with TPC1 being the dominant subtype.^27^ It was revealed that the addition of membrane‐permeant NAADP acetoxymethyl ester in PASMCs induced a biphasic elevation within global [Ca^2+^ ]*i* in an extracellular calcium‐independent way, which could be disrupted via an NAADP antagonist Ned‐19, suggesting the release of calcium from acidic endolysosomal calcium stores.^27^ In addition to the hyperproliferation of pulmonary vascular cells, including PASMC and PAEC, disordered angiogenesis plays an essential role in PAH progression.^9^ Favia et al ^28^ reported a specific calcium signalling associated with the VEGFR2 receptor subtype, regulating the critical angiogenic responses of endothelial cells to VEGF. Interestingly, NAADP engagement and TPC2‐specific involvement in acidic intracellular calcium stores lead to calcium release, and angiogenic responses have been equally mentioned. Although it has been demonstrated that TPC1/TPC2 showed to be expressed within PASMCs of rats, with TPC1 being the dominant subtype, the detailed functions of TPC1/2 in NAADP‐mediated Ca^2+^ mobilization, PASMC proliferation and PAEC angiogenesis are unknown as of yet.

In this study, a hypoxia‐induced rat PAH model was established; PASMCs and PAECs were isolated, and generation of NAADP and TPC1/2 was examined. Furthermore, the effects of TPC1/2 on PAH, on Ca^2+^ with or without NAADP in PASMCs or PAECs, respectively, and on the proliferation of PASMCs or angiogenesis of PAECs were evaluated. This is regarded as a potential target in the treatment of PAH.

## 2 MATERIALS AND METHODS

### 2.1 Hypoxia exposure of animal model

A total of 30 adult male Wistar rats weighing 190‐230 g were exposed to normobaric hypoxia (10% oxygen environment) in a ventilated hypoxia chamber 6 hours daily over 3 weeks and were compared to a control of 30 rats treated in normoxic conditions. To establish a state of hypoxia, the chamber was flushed with a gas mixture of room air and nitrogen from a liquid nitrogen reservoir. The age‐ and weight‐matched control 30 rats were maintained at an oxygen saturation of 21%. The anaesthetic used was sodium pentobarbital at a dosage of 40‐60 mg/kg. CO_2_ inhalation was used to achieve euthanasia. The aforementioned mentioned process was carried out under the administration of an analgesic. One week following exposure, all rats were injected with lentivirus containing sh‐NC, sh1‐TPC1, or sh2‐TPC1 or sh1‐TPC2 or sh2‐TPC2. A further two weeks later, the pressure of the right ventricle (RV) and left ventricle (LV) was measured; the pulmonary tissues were harvested for additional assays. The pulmonary artery was infused with normal saline to cleanse the blood off of the pulmonary vessels. For histological analysis, the pulmonary veins were subsequently ligated. The pulmonary artery and trachea were perfused with 10% paraformaldehyde at constant pressure (100 cm H_2_O for the pulmonary artery and 25 cm H_2_O for the trachea) to fully distend the pulmonary blood vessels and airway, respectively. Peripheral lung specimens were then excised, fixed in 10% paraformaldehyde, paraffin‐embedded and sectioned for morphometric analysis of pulmonary arterioles. All vivisection experiments were performed as per National Institutes of Health (NIH) guidelines and were approved by the institutional standing committee on animals. The flow chart of animal model building and further experiments is depicted in Figure S1.

### 2.2 Haematoxylin and eosin (H&E) staining

Frozen sections were warmed for 60 minutes at room temperature, fixed for 15 minutes in the precooled acetone at 4°C and rinsed for 3 minutes with distilled water. The sample was stained for 5 minutes in the haematoxylin staining solution (Beijing Beifang Biological Technology Research Institute) and then rinsed once with water before being placed in 95% ethanol for 5 seconds and stained for approximately 1 minutes in the eosin solution (Beijing Beifang Biologic Technology Research Institute). The sample was then immersed in 95% ethanol for 2 minutes, transferred to xylene, mounted with central gum and observed under a microscope (Takara, Bio, Inc). The nucleus was stained in blue, whereas the cytoplasm was red or lighter hues of red. Photographic analysis was carried out with an inverted microscope. The wall thickness was quantified and analysed using the following formula: ratio = (external diameter (ED)‐inner diameter (ID))/ED

### 2.3 ELISA assay for NAADP

The NAADP concentrations in lung tissues were determined through the enzyme‐linked immunosorbent (ELISA) assay kit purchased from MyBioSource (Catalog No: MBS452979). The operation was carried out as per the instructions manual of the ELISA kit. The 96‐well plates were briefly covered with 100 μl/well of capture antibodies for each cytokine, as per the manufacturer's instructions (Peprotech Inc). The plates were incubated overnight at room temperature and washed with 0.05% Tween‐20 in PBS thrice. Then, 300 μl of blocking buffer 1% BSA in PBS was added to each well. Plates were incubated for 1 hour, and 100 μl of the samples (diluted 1:2) or standard solutions was added. The plates were incubated at room temperature for 2 hours. 100 μl of detection antibody and 100 μl of a conjugated avidin solution were added to each well. After 20 minutes, 100 μl of ABTS liquid substrate solution was added. The plates were maintained at ambient temperature, and the colorimetric reaction was read in a spectrophotometer at a wavelength of 405 nm with a correction of 650 nm.

### 2.4 Isolation of rat PASMCs

The isolation of rat PASMCs was in accordance with previously carried out studies.^29^ Rat PASMCs were isolated from male Wistar rats. The anaesthetic used was sodium pentobarbital at a dosage of 60 mg/kg. Subsequently, PASMCs were enzymatically isolated and transiently cultured in DMEM for 3 passages for stable cultivation in vitro. The isolated PASMC cells were identified by immunofluorescence using the antibody against α‐SMA protein antibody (#ab5694, Abcam). The experiment was performed on the third passage cells from the same batch of isolated PASMC. Exposure of cells to chronic hypoxia was performed by transferring them to an incubator gassed with 2.5% O_2_, 5% CO_2_ and 92.5% N_2_ for 24 hours before the study.

### 2.5 Isolation of rat PAECs

The isolation of rat PAECs was according to the previous studies.^30^ Animals were euthanized with 60 mg/kg intraperitoneal sodium pentobarbital. A 14‐gauge angiocatheter was inserted into the trachea through a midline neck incision and secured with a 4‐0 braided silk suture. A median sternotomy was performed, and the heart‐lung block was rapidly excised. Endotracheal lavage of the lungs was performed 15 times with 6‐9 ml phosphate‐buffered saline (PBS) containing 0.25 mmol/L EDTA to deplete alveolar macrophages.^31^ 2 mm strips of peripheral lungs were excised from all lung lobes. Tissue was minced, rinsed in RPMI media, transferred to a dispase (10 mg/ml) solution and then incubated for 60 minutes at 37°C. The cell suspension was homogenized and incubated for a further 5 minutes at 37°C. A total of 10 ml of complete media containing 10% foetal bovine serum (FBS) was added to terminate the reaction, and the cellular suspension was then filtered through a 100 μm mesh. The filtrate was spun at 800 g for 8 minutes, resuspended in supplemented RPMI media and plated on gelatin‐coated culture dishes. The media was changed every 48 hours during the incubation period until confluence.^32^ Cells were labelled with Ac‐LDL and were separated using flow cytometry (FAC STAR Plus), which maintained a pure culture of endothelial cells. The isolated PAECs show CD31, von Willebrand factor (vWF) and VEGFR2‐positive (Figure S3F). All cells used in these experiments were from 3 passages. Exposure of cells to chronic hypoxia was performed by transferring them to an incubator gassed with 2.5% O_2_, 5% CO_2_ and 92.5% N_2_ for 24 hours before study.

### 2.6 RNA preparation and real‐time PCR

Total RNA was extracted from lung tissues and primary cells using the RNeasy mini kit (Qiagen) following standard procedures. Real‐time PCR was performed on triplicate samples using a miScript SYBR Green PCR kit (Qiagen) on the ABI 7500 Real‐time PCR System (Applied Biosystem, USA) following the 2^−ΔΔCt^ method.

### 2.7 TPC1/2 shRNA lentivirus construction and infection

An effective short hairpin RNA targeting rat TPC1 or TPC2 was constructed and subcloned into the plasmid of lentivirus vectors, pLKO.1‐EGFP. Both the pLKO.1‐EGFP‐TPC1/2 and Lentivector package plasmid mixes were transferred into the 293T cells (ATCC, USA). The culture supernatant was harvested, and the virus titre was detected 48 hours following transfer. Rats were randomly divided into 2 groups: the normoxia group and hypoxia group. The rats in both hypoxia and normoxia group underwent venous injection of Lsh‐NC (negative control), Lsh1‐TPC1 or Lsh2‐TPC1 or Lsh1‐TPC2 or Lsh2‐TPC2 lentivirus (500 μl, 1 × 10^8^ TU/ml) on the 7th day after hypoxia treatment. A fortnight later, the contents of TPC1/2 mRNA and TPC1/2 protein in lung tissue were detected through real‐time PCR and Western blot, respectively. For cell transfection, rat PASMCs or PAECs were plated at a concentration of 2 × 10^5 ^cells/ml and incubated for 16 hours. For infection, 1.5 ml/well viral supernatant was used to replace the medium. Cells were incubated at 37°C for 10 hours, and then, the fresh media was used to replace the viral supernatant. 48 hours after the infection, the infection efficiency was verified using Western blot or real‐time PCR assays.

### 2.8 Blocking of Ca^2+^ flux

The specific TPC blocker Ned‐19 and calcium channel inhibitor tetrandrine (all purchased from Sigma) were used for Ca^2+^ blocking as the previous method described.^33^, ^34^ Briefly, PASMCs and PAECs were pretreated with 200 μmol/L Ned‐19 for 30 minutes or 100 μmol/L tetrandrine for 5 minutes.

### 2.9 Western blotting

Primary cultures of rat PASMCs and PAECs were washed with phosphate‐buffered saline. The homogenate was centrifuged at 1000 × *g* for 5 minutes at 4°C, the resulting supernatant was subsequently collected, and the protein concentration was estimated using a BCA assay. 20 μg of protein sample was resolved on an 8% SDS‐polyacrylamide gel and electro‐transferred onto a PVDF membrane (Millipore). The membrane was blocked with 5% (w/v) nonfat dry milk in PBS containing 0.02% Tween‐20 for 1 hours at room temperature, followed by overnight incubation at 4°C with a specific primary antibody. The following antibodies were used: anti‐TPC1 (ab94731, Abcam), anti‐TPC2 (ab119915, Abcam), anti‐CaMKII (Cat#6G9, Abcam), anti‐calcineurin (Cat#EP1669Y, Abcam) and anti‐GAPDH (Cat#6C5, Abcam). The GAPDH level was also determined and used as a loading control. The membrane was washed and incubated with the peroxidase‐conjugated goat anti‐rabbit secondary antibody (Bio‐Rad) at 1:2500 dilution at room temperature for 1 hours. Protein bands were detected by enhanced chemiluminescence (Pierce) and photographed using a Gel Logic 200 image system (Kodak).

### 2.10 Calcium imaging

For intracellular Ca^2+^ ([Ca^2+^] *_i_*) determination, PASMCs or PAECs cultured on 35‐mm dishes were incubated in a culture medium containing 3.5 μmol/L FURA‐2‐AM (Invitrogen) for 1 hours at 37°C and subsequently rinsed with HBSS (Sigma). Each dish was placed into a culture chamber at 37°C on the stage of an inverted fluorescence microscope (NikonTE2000E), connected to a cooled CCD camera (512B Cascade, Roper Scientific). Samples were illuminated alternately at 340 and 380 nm using a random‐access monochromator (Photon Technology International), and emission was detected using a 510 nm emission filter. Images were acquired (1 ratio image per second) using Metafluor software (Universal Imaging Corporation).

### 2.11 MTT assays

Cells were plated in a 96‐well plate at 2 × 10^4^ per well and cultured for 24 hours, followed by infection of Lsh‐TPC1 in the absence or presence of NAADP (Cat No: 21 000, AAT Bioquest). The MTT (Sigma) was added until a final concentration of 0.5 mg/ml, and the cells were incubated for 4 hours at 37°C. The absorbance at 490 nm was measured by a microplate reader (Bio‐Rad). Each experiment was repeated at least thrice in triplicate.

### 2.12 BrdU incorporation assay

By measuring 5‐bromo‐2‐deoxyuridine (BrdU) incorporation, the DNA synthesis in proliferating cells was determined. Cells were seeded in 96‐well culture plates at a density of 2 × 10 cells/well and infected with Lsh‐TPC1 in the absence or presence of NAADP. Following a period of 24 hours or 48 hours, cells were incubated with a final concentration of 10 μmol/L BrdU (BD Pharmingen) for 2 hours. Then, the medium was removed, the cells were incubated with peroxidase‐coupled anti‐BrdU antibody (Sigma‐Aldrich) for 60 minutes at RT, washed three times with PBS, incubated with peroxidase substrate (tetramethylbenzidine) for 30 minutes, and the 450 nm absorbance values were measured for each well. Background BrdU immunofluorescence was determined in cells not exposed to BrdU but stained with the BrdU antibody.

### 2.13 Statistical analysis

Data are expressed as means ± SD Statistical significance (*P* < .05) of the changes was assessed by paired or unpaired Student's *t* tests.

## 3 RESULTS

### 3.1 Identification of hypoxia‐induced rat PAH model

**FIGURE 1:**
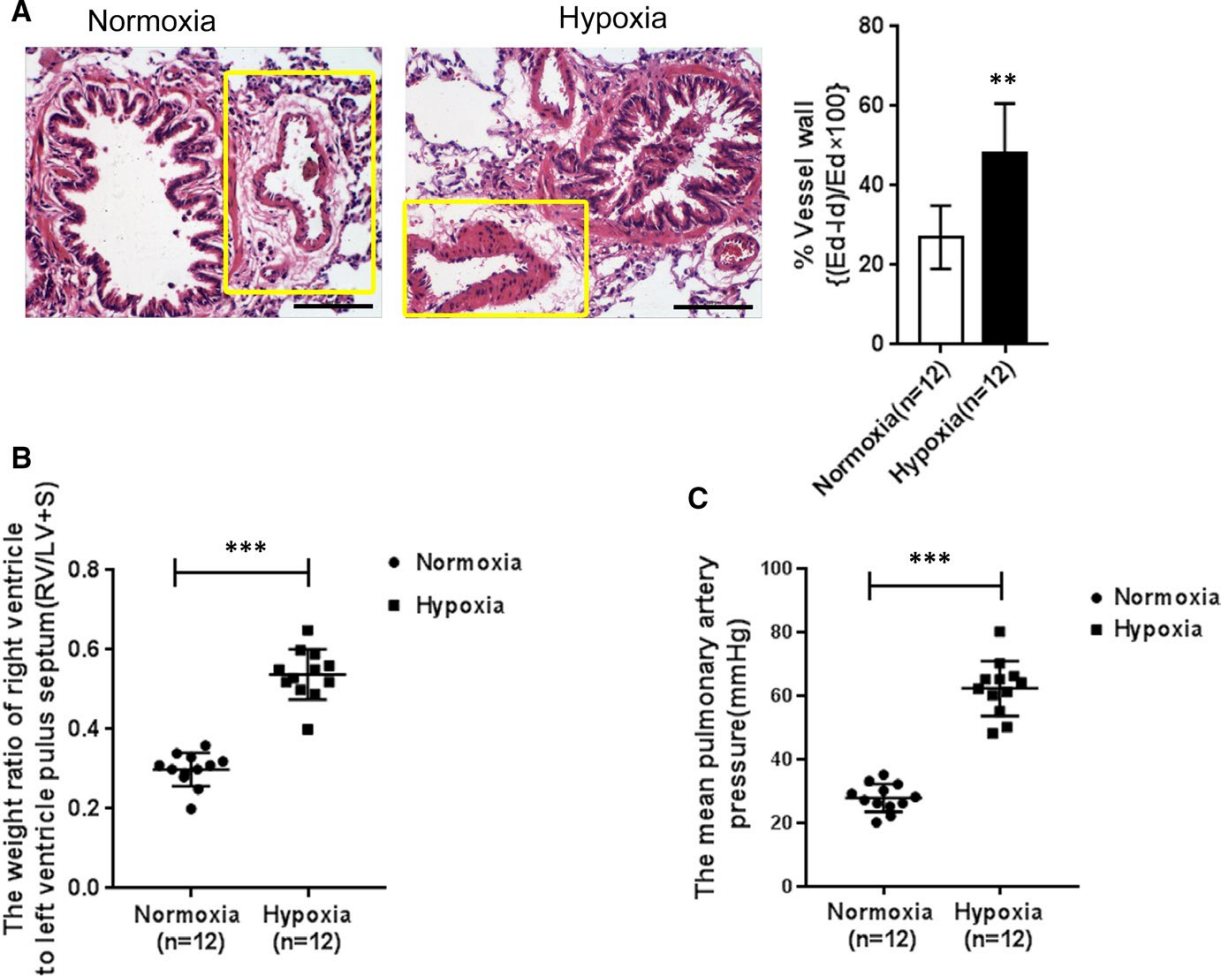
Identification of hypoxia‐induced rat PAH model. In the chronic hypoxic exposure‐induced PAH model group (n = 12) and normoxia control group (n = 12) (A) H&E staining: the mean arterial wall thickness is shown as the ratio of (external diameter (ED)‐inner diameter (ID))/ED (scale bar is 100 μm). The yellow boxes indicated pulmonary artery. (B) and (C) mean pulmonary artery pressure (B) and RV/LV + S ratio (C) were determined and used to assess the model development. The experiments were repeated for at least 3 times. The aster‐marks *indicated the statistic different value *P* < .05, and ***indicates *P* < .001

The chronic hypoxic exposure‐induced PAH model development was assessed by measuring the pulmonary artery wall thicknesses through H&E staining the average pulmonary artery pressure was measured by right ventricular catheterization and left/right ventricular weight ratio (RS/LV + S). H&E staining results showed that following hypoxia exposure, the pulmonary arterial wall was thickening while narrowing its lumen, manifested as the up‐regulation of the external diameter ratio (ED): inner diameter (ID) by hypoxia stress. (Figure 1A); the mean pulmonary arterial pressure (mPAM) in the hypoxia exposure group showed to be markedly increased as compared to that from the normal group (*P* < .01) (Figure 1B). The RS/LV + S from each group was also calculated. The model group also has a much higher ratio of RS/LV + S compared with the normal group (*P* < .01) (Figure 1C). The aforementioned pulmonary artery alterations indicated that the model of vascular PAH induced by hypoxia was successfully established.

### 3.2 NAADP generation and its correlation with TPC1/2 under normoxia or hypoxia condition

**FIGURE 2:**
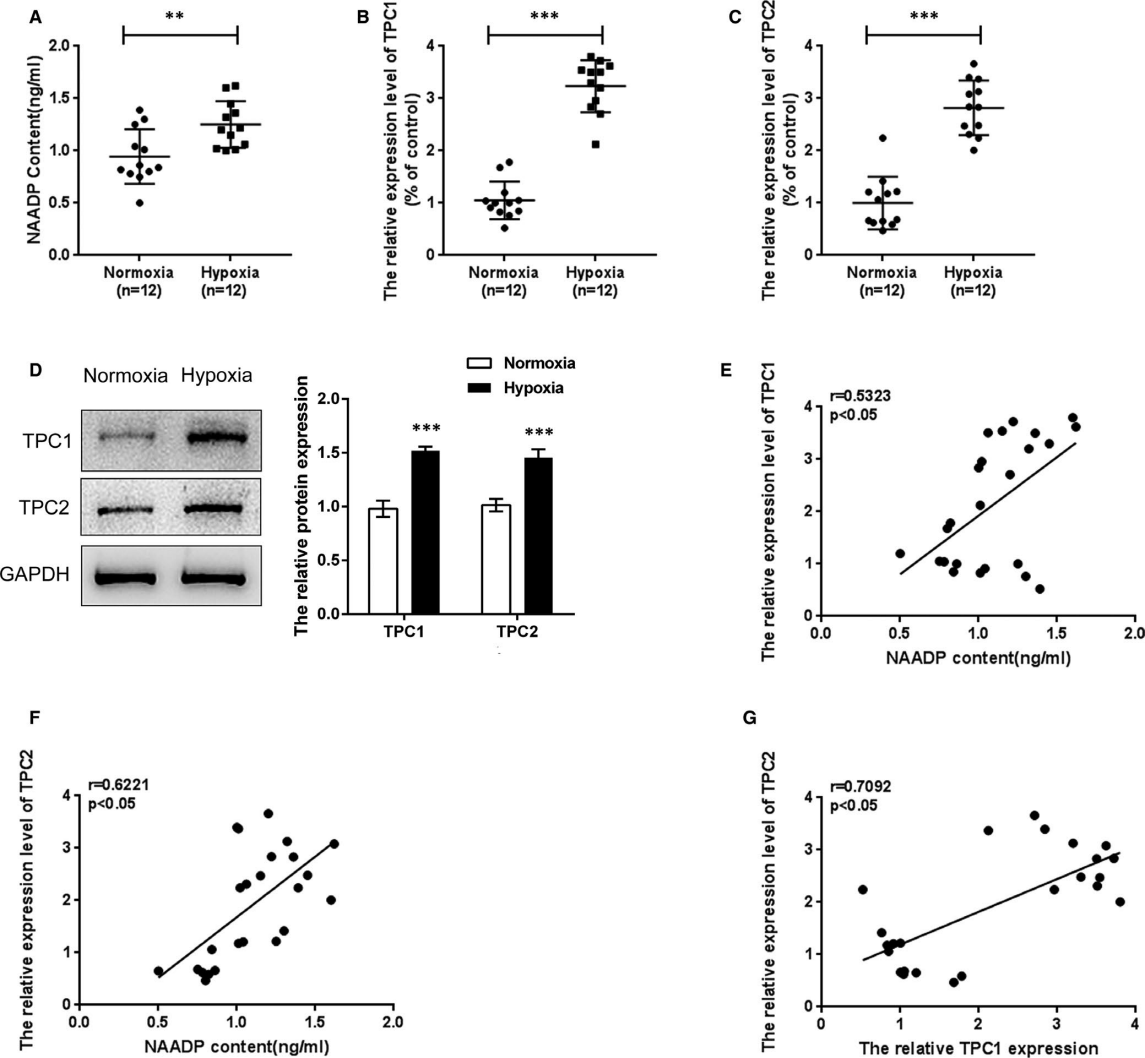
NAADP generation and its correlation with TPC1/2 under normoxia or hypoxia condition (A) The NAADP content in PAH rat lung tissues was determined using ELISA assays, compared with normal tissues. (B)‐(D) The mRNA levels and protein levels of TPC1/2 were detected using real‐time PCR and Western blot assays. The experiments were repeated for at least 3 times. The aster‐marks *indicated the statistic different value *P* < .05, and ***indicates *P* < .001. (E),(F). The correlations of NAADP, TPC1 and TPC2 were analysed using Spearman's rank correlation analysis

To explore the possible role of TPC1/2 in the pathogenic process of hypoxic exposure‐induced PAH, the NAADP concentration, TPC1/2 mRNA and protein expression within PAH rat lung tissues in each group were assessed and their correlations were analysed. ELISA results showed that NAADP concentration in the hypoxic group was significantly up‐regulated when compared to the normoxia group (Figure 2A). Consistent with NAADP concentration changes, the mRNA and protein levels of TPC1 and TPC2 also showed to be remarkably increased in the hypoxic group compared with the normoxia group (Figure 2B‐D). Besides, Spearman's rank correlation analysis was performed to confirm that NAADP content had a positive correlation with TPC1/2 expression, respectively; TPC1 expression also had a positive correlation with TPC2 expression (Figure 2E‐G). The above‐mentioned results indicated the potential roles of NAADP and TPC1/2 in the hypoxia‐induced rat pulmonary artery hypertension model.

**FIGURE 3:**
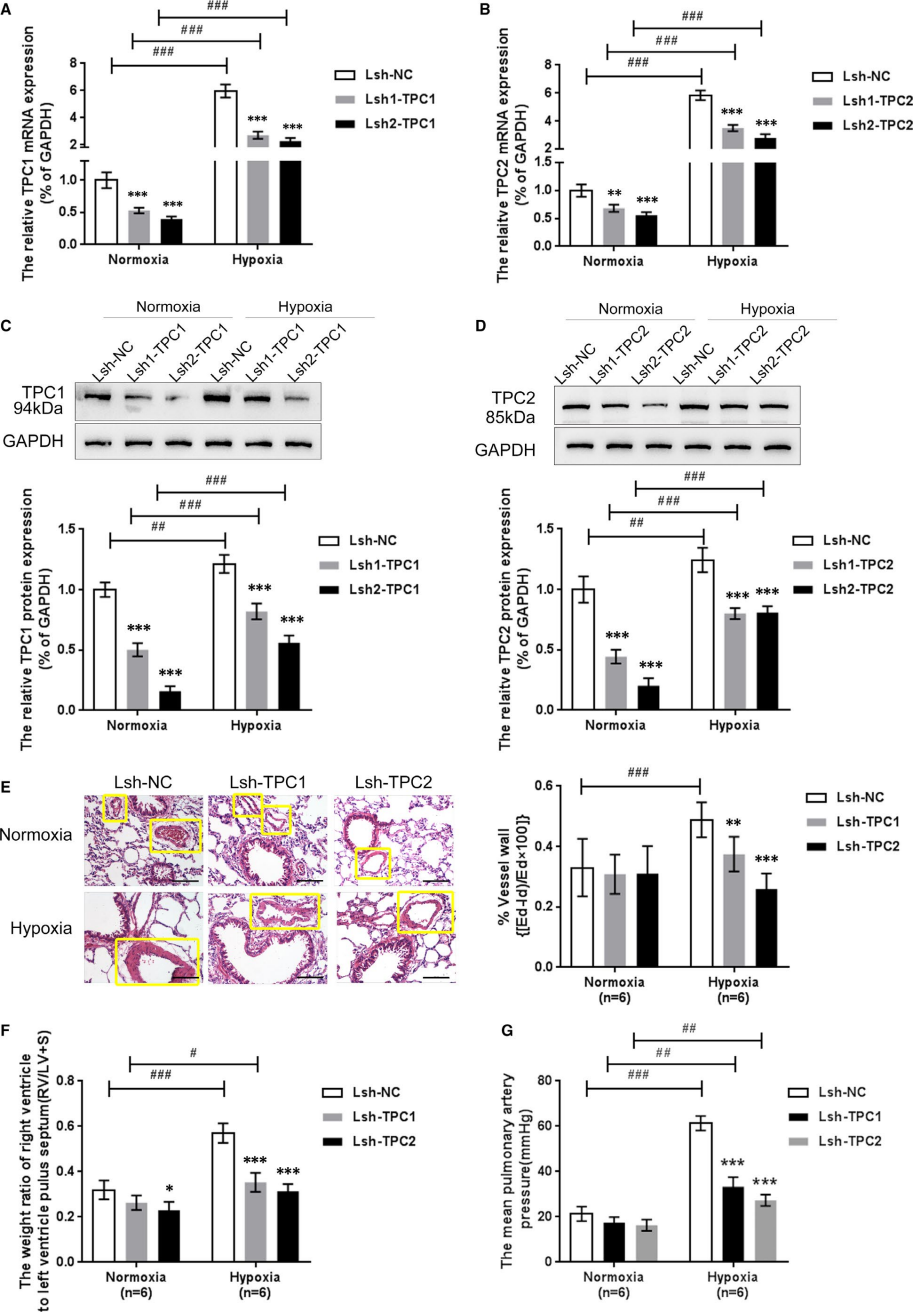
Effects of TPC1/2 on hypoxia‐induced rat PAH model (A)‐(D) Lsh1‐TPC1 or Lsh2‐TPC1 or Lsh1‐TPC2 or Lsh2‐TPC2 infected to the PAH rat to achieve TPC1/2 knockdown; the mRNA expression and protein levels of TPC1/2 were first verified using real‐time PCR and Western blot assays before infection. (E) The changes in pulmonary morphology in peripheral lung sections from the hypoxic group and normoxic control animals by H&E staining (Scale bar is 100 μm). The yellow boxes indicated pulmonary artery. (F)‐(G)the mPAP (mean pulmonary artery pressure) (F) and the RS/LV + S (G) were dramatically up‐regulated under hypoxia condition, compared with normoxia condition, whereas partially declined by Lsh‐TPC1 or Lsh‐TPC2 treatment than those from hypoxia + Lsh‐NC group. The marks *indicates the significant different value *P* < .05, **indicates *P* < .01, and ***indicates *P* < .001; the marks #indicates the significant different value *P* < .05, ##indicates the significant different value *P* < .01, and ###indicates the significant different value *P* < .001

In order to further explore the possible functional roles of TPC1/2 in PAH, a lentiviral vector expressing shRNA targeting TPC1/2 was constructed, namely (Lsh1‐TPC1, Lsh2‐TPC1, Lsh1‐TPC2 and Lsh2‐TPC2) and the negative control lentiviral vector (Lsh‐NC containing the coding sequence for nonsense siRNA). Rats in hypoxia and normoxia groups were injected with the above lentivirus, respectively. Real‐time PCR was performed to verify the inhibitory efficiency of TPC1/2 mRNA expression in PAH rat lung tissues, which demonstrated that Lsh1‐TPC1/ Lsh2‐TPC1 significantly down‐regulated TPC1 mRNA expression (Figure 3A‐B), and Lsh1‐TPC2 and Lsh2‐TPC2 significantly down‐regulated TPC2 mRNA expression (Figure 3A‐B). A Western blot was performed to verify the inhibitory efficiency of TPC1/2 proteins; similar to mRNA expression, Lsh1‐TPC1 and Lsh2‐TPC1 caused a dramatic decrease in TPC1 protein, whereas Lsh1‐TPC2 and Lsh2‐TPC2 caused a dramatic reduction in TPC2 protein (Figure 3C‐D). Lsh2‐TPC1 and Lsh2‐TPC2 caused a more noticeable change in TPC1 or TPC2 mRNA and protein expression, respectively, thus were selected as shRNA for TPC1 or TPC2 for further experiments.

To further explore the possible role of TPC2 in PAH, the changes in pulmonary morphology in peripheral lung sections from the hypoxic group and normoxic control animals were examined by H&E staining (Figure 3E). Results showed that the normal group had clear boundaries of the pulmonary artery and structural integrity, whereas the model group had a destructed and blurred boundary of the artery; under chronic hypoxic exposure condition, either TPC1 or TPC2 shRNA injection group apparently had better clearer boundaries and structural integrity than the control group (Lsh‐NC) (Figure 3E). Moreover, the thickness of the arterial wall (ratio = (ED‐ID)/ED) was significantly increased under hypoxic conditions but was remarkably reduced by Lsh‐TPC1 or Lsh‐TPC2 (Figure 3E). These results showcased the histopathological changes during pulmonary artery hypertension.

To confirm the possible role of TPC1/2 in PAH, the mean pulmonary artery pressure and the RS/LV + S from each group were calculated. The result proved that both the mPAP (mean pulmonary artery pressure) (Figure 3F) and the RS/LV + S (Figure 3G) were dramatically up‐regulated under hypoxic conditions, compared with normoxic conditions. In contrast, it was partially declined by Lsh‐TPC1 or Lsh‐TPC2 treatment than those from hypoxia + Lsh‐NC group (*P* < .01). Interestingly, TPC1/2 knockdown has not induced any apparent reversal in pulmonary artery pressure and RS/LV + S under normoxic conditions, suggesting that TPC1/2 exert their effect under hypoxic stress. The above data prove that the TPC1/2 knockdown could partially improve hypoxia stress‐caused changes during PAH.

### 3.3 The effect of TPC1/2 on [Ca^2+^]_i_ in PASMCs

In our previous study, it was demonstrated that NAADP‐mediated Ca^2+^ signals in PASMCs between endolysosomes and the sarcoplasmic reticulum modulate vascular reactivity and other cell functions. Herein, it is revealed that TPC1/2 knockdown proved the mean pulmonary artery pressure and PAH morphology. To further investigate the mechanism by which TPC1/2 might affect PAH development, the effects of TPC1/2 on [Ca^2+^]_i_ in PASMCs were subsequently evaluated. Firstly, the impact of hypoxia on NAADP‐mediated Ca^2+^ signals was validated in PASMCs and PAECs. Figure S2 and Figure S3 show that NAADP‐AM‐mediated sharp increases in [Ca^2+^]_i,_ and Ca^2+^ signal markers were enhanced in PAECs and PASMCs under hypoxia, compared with normoxic group. Moreover, under nigericin (50 μmol/L) treatment, which disrupted the lysosomal Ca^2+^ store,^35^ the [Ca^2+^]*_i_* was increased in PAECs and PASMCs in response to hypoxia, indicating that the lysosomal Ca^2+^ content was increased in the hypoxic group (Figure S2E and Figure S3E). Besides, TPC1/2 proteins within PASMCs and PAECs showed to be examined under hypoxia. Figure S4 shows that hypoxia dramatically increased TPC1/2 in both cell lines.

**FIGURE 4:**
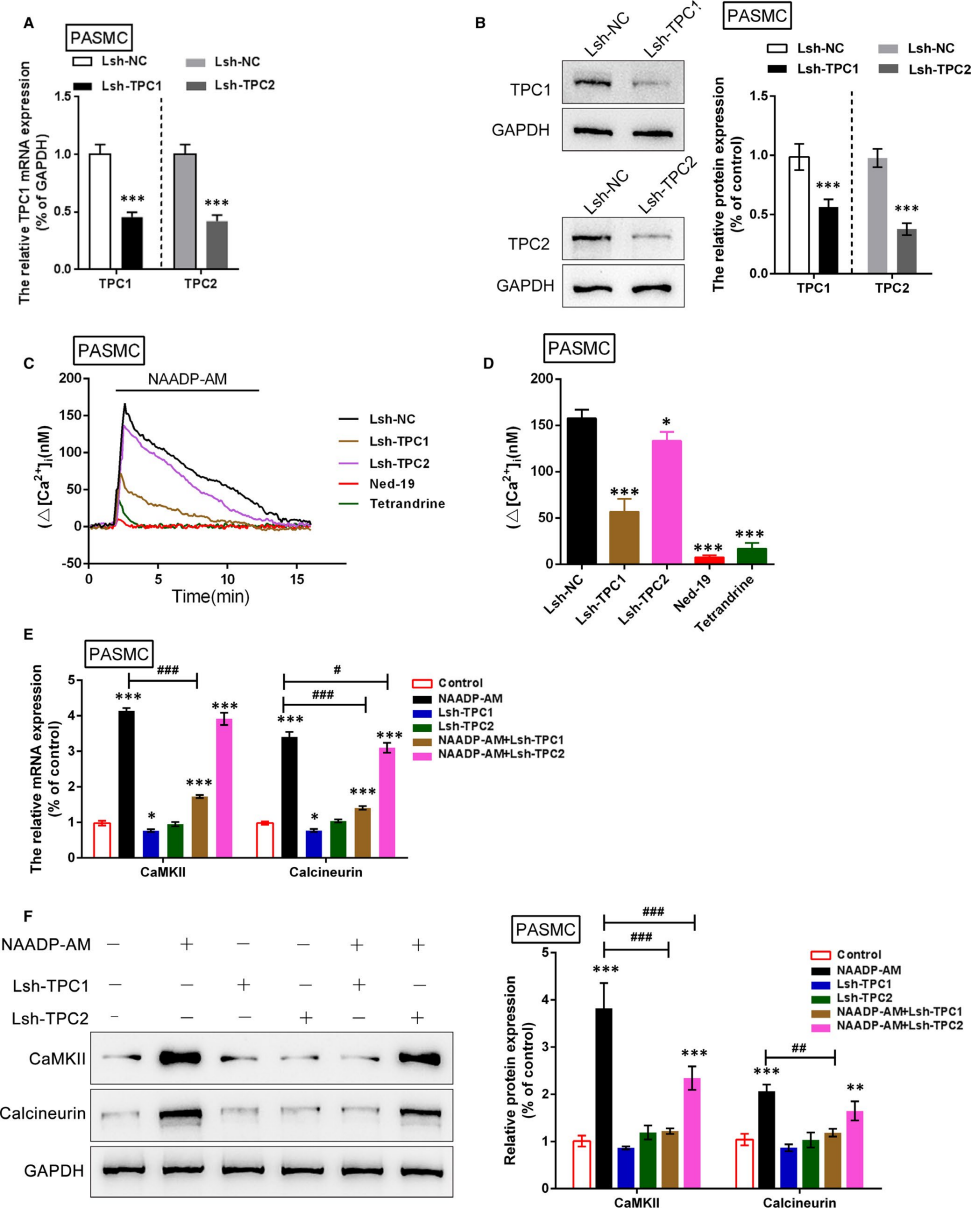
Effects of TPC1/2 on Ca mobilization in PASMCs (A)‐(B) PASMCs were infected with Lsh‐TPC1 or Lsh‐TPC2 to achieve TPC1 or TPC2 knockdown, as verified using real‐time PCR and Western blot assays. (C) and (D) [Ca^2+^]*_i_* in TPC blockers‐treated, Lsh‐TPC1‐ or Lsh‐TPC2‐infected PASMCs with the NAADP‐AM stimulation was determined. (E) and (F) The mRNA and protein levels of CaMKII and calcineurin in Lsh‐TPC1‐ or Lsh‐TPC2‐infected PASMCs in the presence or absence of NAADP‐AM stimulation were determined using real‐time and Western blot assays. The experiments were repeated for at least 3 times. The marks *indicates the significant different value *P* < .05, **indicates *P* < .01, and ***indicates *P* < .001 compared with control group; the marks #indicates the significant different value *P* < .05, ##indicates the significant different value *P* < .01, and ###indicates the significant different value *P* < .001 compared with NAADP‐AM group

Real‐time PCR and Western blot were performed to verify that PASMCs were infected with Lsh‐TPC1/2 to achieve TPC1/2 knockdown (Figure 4A and B). The [Ca^2+^]_i_ was then determined in infected PASMCs. Results showed that Lsh‐TPC1 and Lsh‐TPC2 infection or treatment with TPC blocker Ned‐19 or calcium channel inhibitor tetrandrine caused significant suppression on NAADP‐AM‐activated [Ca^2+^]_i_, compared with Lsh‐NC group; the effect of TPC1 knockdown was close to that of Ned‐19 and tetrandrine treatment (Figure 4C,D).

The changes in mRNA and protein expression of Ca^2+^ signalling‐related factors, CaMKII and calcineurin, in response to co‐processing NAADP‐AM and TPC1/2 knockdown were further monitored. Consistent with [Ca^2+^]_i_ changes, CaMKII and calcineurin mRNA and protein expression could be significantly enhanced via NAADP‐AM treatment, whereas it was inhibited via TPC1 silencing; TPC1/2 silence might significantly attenuate how NAADP‐AM promoted CaMKII and calcineurin expression, which showed to be more strongly attenuated by TPC1 silence (Figure 4E,F). In summary, TPC1/2 could potentially affect the Ca^2+^ signalling in PASMCs, with TPC1 playing the dominant role.

### 3.4 The effects of TPC1 on PASMC proliferation

**FIGURE 5:**
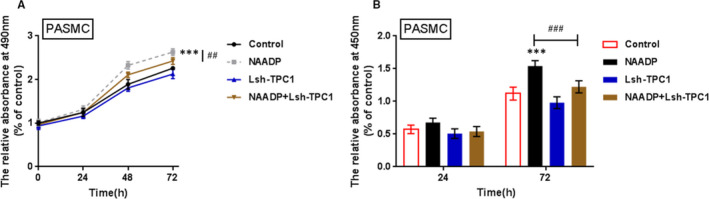
Effects of TPC1 on PASMC proliferation (A) and (B) PASMCs were infected with Lsh‐TPC1 with the presence or absence of NAADP stimulation; the proliferation of PASMC was determined using MTT (A) and BrdU assays (B). The experiments were repeated for at least 3 times. The marks *indicates the significant different value *P* < .05, and ***indicates *P* < .001 compared with control group; the marks ##indicates the significant different value *P* < .01, and ###indicates the significantly different value *P* < .001 compared with NAADP group

As it has been proven that TPC1/2 could affect Ca^2+^ signalling in PASMCs, with TPC1 playing the dominant role, the effects of TPC1 on regulating the proliferation of PASMCs were further evaluated to confirm the dominant role of TPC1 in PASMCs. NAADP induced PASMC proliferation, whereas TPC1 knockdown alone did not cause an apparent change in the proliferation of PASMCs; TPC1 knockdown could significantly attenuate how NAADP promoted the proliferation of PASMCs (Figure 5A,B); it was further confirmed that TPC1 could inhibit Ca^2+^ signalling in PASMCs and PASMC proliferation to prove PAH, but caused no noticeable change in PASMC proliferation alone.

### 3.5 The effect of TPC1/2 on [Ca^2+^]_i_ in PAECs

**FIGURE 6:**
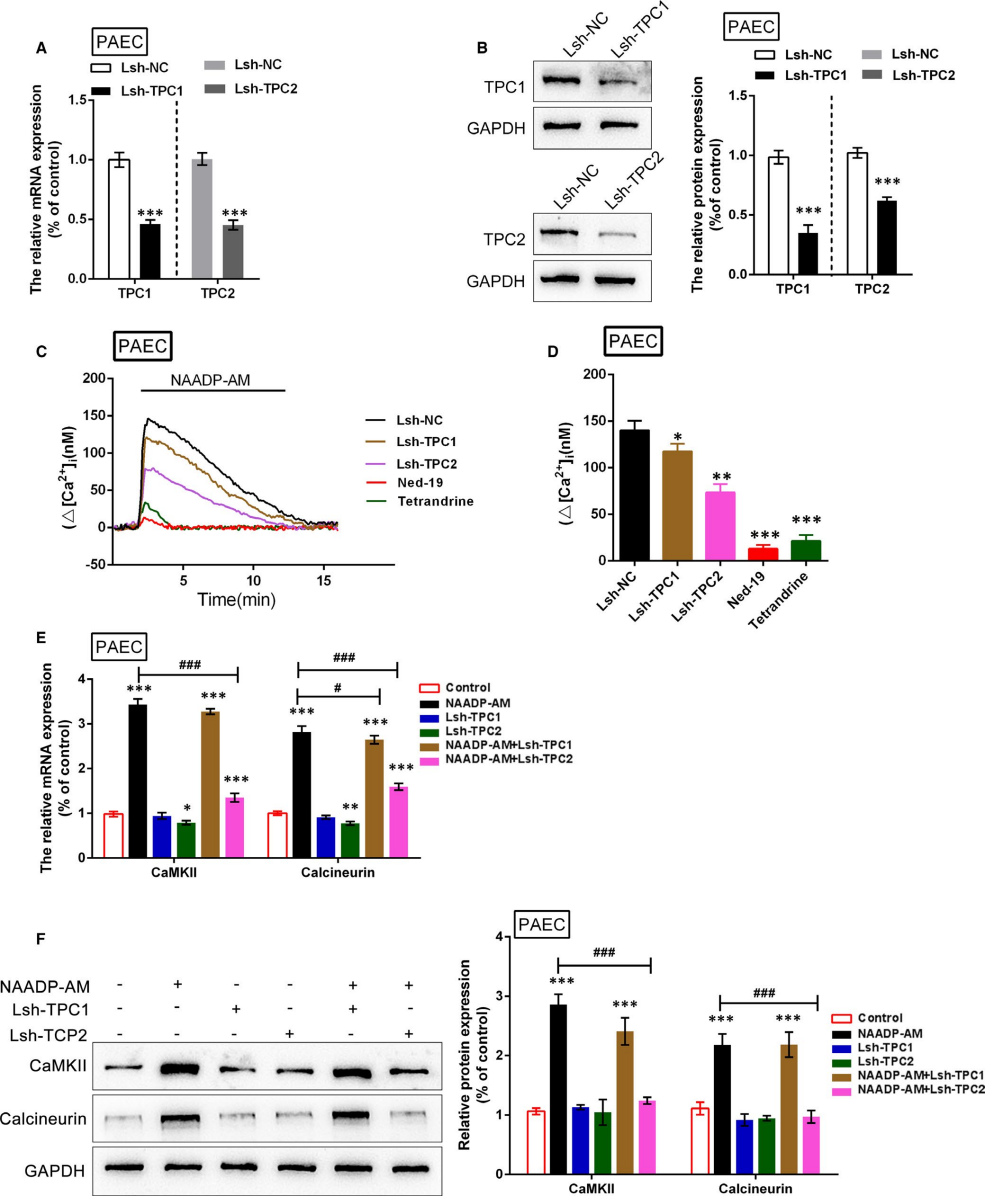
Effects of TPC1/2 on Ca mobilization in PAECs (A)‐(B) PAECs were infected with Lsh‐TPC1 or Lsh‐TPC2 to achieve TPC1/2 knockdown, as verified using real‐time PCR and Western blot assays. (C) and (D) [Ca^2+^]*_i_* in TPC blockers‐treated, Lsh‐TPC1‐ or Lsh‐TPC2‐infected PAECs in the presence of NAADP‐AM stimulation were determined. (E) and (F) the mRNA and protein levels of CaMKII and calcineurin in Lsh‐TPC1‐ or Lsh‐TPC2‐infected PAECs in the presence or absence of NAADP‐AM stimulation were determined using real‐time and Western blot assays. The experiments were repeated for at least 3 times. The marks *indicates the significant different value *P* < .05, **indicates *P* < .01, and ***indicates *P* < .001 compared with control group; the marks #indicates the significant different value *P* < .05, ##indicates the significant different value *P* < .01, and ###indicates the significant different value *P* < .001 compared with NAADP‐AM group

It has been demonstrated that TPC1/2 could affect NAADP‐AM‐mediated Ca^2+^ signalling in PASMCs upon hypoxia exposure, with TPC1 playing the dominant role; we further investigated the functions of TPC1/2 in PAECs were further investigated PAECs were infected with Lsh‐TPC1/Lsh‐TPC2 to achieve TPC1/2 knockdown (Figure 6A,B). The indicated assays were performed in a similar fashion in PAECs to evaluate the effects of TPC1/2 on Ca^2+^ mobilization in PAECs. Consistent with the earlier results, the addition of NAADP‐AM immediately caused a prominent elevation within [Ca^2+^]_i_, whereas TPC1/2 knockdown alone caused no significant changes in [Ca^2+^] _i_. Interestingly, unlike the previous results, the promotive effect of NAADP‐AM on [Ca^2+^] _i_ could be reversed by TPC1 and TPC2 knockdown or treatment with tetrandrine or Ned‐19; the effect of TPC2 knockdown was close to that of tetrandrine and Ned‐19 treatments (Figure 6C,D). Subsequent determination of CaMKII and calcineurin expression yielded consistent results: NAADP‐AM significantly increased CaMKII and calcineurin mRNA and protein expression; the promotive role of NAADP‐AM could be slightly declined by the TPC1 knockdown but could be significantly reversed by TPC2 knockdown (Figure 6E,F). In summary, TPC1/2 might modulate NAADP‐AM‐mediated Ca^2+^ mobilization in PAECs, with TPC2 playing the dominant role.

### 3.6 The effects of TPC2 on angiogenesis in PAECs

**FIGURE 7:**
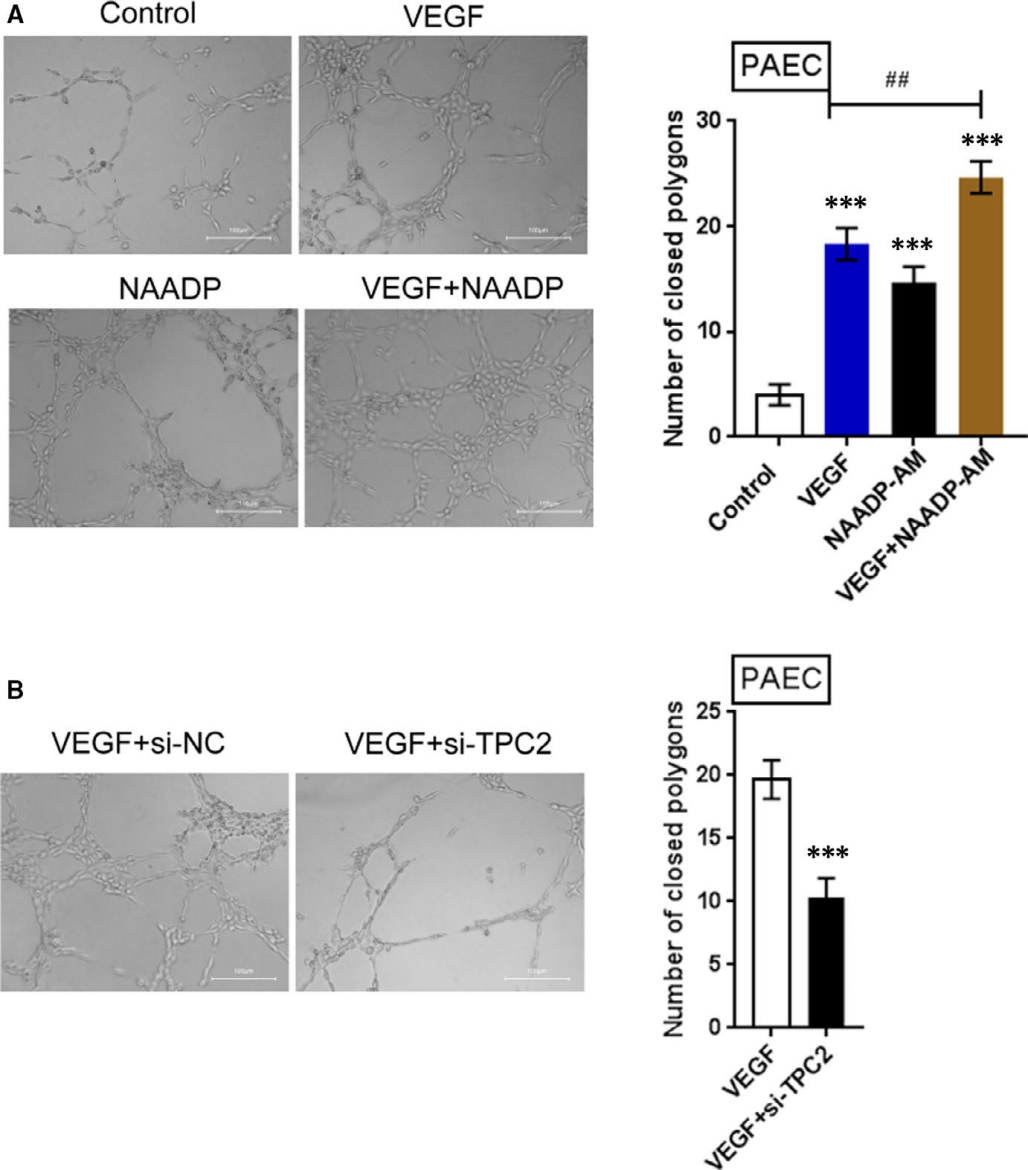
Effects of TPC2 on angiogenesis in PAECs (A) The effects of VEGF and/or NAADP on angiogenesis of PAEC; quantitative evaluation of tube formation as the number of closed polygons formed in five fields for each experimental condition. (B) The effects of TPC2 knockdown on angiogenesis of PAEC; quantitative evaluation of tube formation as the number of closed polygons formed in five fields for each experimental condition

In PAH, the functions of TPC2 on angiogenesis in PAECs were further evaluated to confirm the dominant role of TPC2 in PAECs. VEGF (vascular endothelial growth factor) exerts a significant effect on controlling the formation of blood vessels.^36^ Stimulating endothelial cells with VEGF has been reported to promote IP3 production and activate the endothelial Ca^2+^signals.^27^ Moreover, NAADP‐induced TPC‐mediated Ca^2+^ release is critical for VEGF‐induced angiogenesis.^26^, ^28^ To investigate the possibility that NAADP was involved in PAEC angiogenesis, PAECs were stimulated with VEGF and NAADP; the angiogenesis of PAEC was subsequently monitored. Results showed that the inducible effect of VEGF on PAEC angiogenesis was amplified by NAADP stimulation (Figure 7A). Figure 7A shows that the number of closed polygonal structures formed within VEGF‐stimulated or NAADP‐AM‐stimulated cells was dramatically elevated compared with that from the control group; the promotive effect of VEGF on closed polygon number was enhanced by NAADP‐AM (Figure 7A). These findings indicate that the NAADP‐AM‐induced calcium signalling pathway was involved in the formation of capillary‐like structures in vitro. PAECs were then infected with Lsh‐TPC2; the angiogenesis of PAEC was subsequently evaluated. Following TPC2 knockdown, VEGF‐induced PAEC angiogenesis significantly declined (Figure 7B); quantitative evaluation also showed that the number of closed polygonal structures formed within si‐TPC2‐transfected cells was significantly reduced, compared with the single VEGF stimulation group (Figure 7B). Furthermore, a VEGF‐induced increase in [Ca^2+^]*_i_* could be suppressed by TPC2 silence (Figure S5). These data indicate that TPC2 silence could potentially inhibit Ca^2+^ signalling in PAECs and PAEC angiogenesis to prove PAH.

### 3.7 A schematic diagram exhibiting NAADP‐induced Ca^2+^ release is mediated by TPC1/2 in hypoxia‐induced PAH (pulmonary arterial hypertension)

**FIGURE 8:**
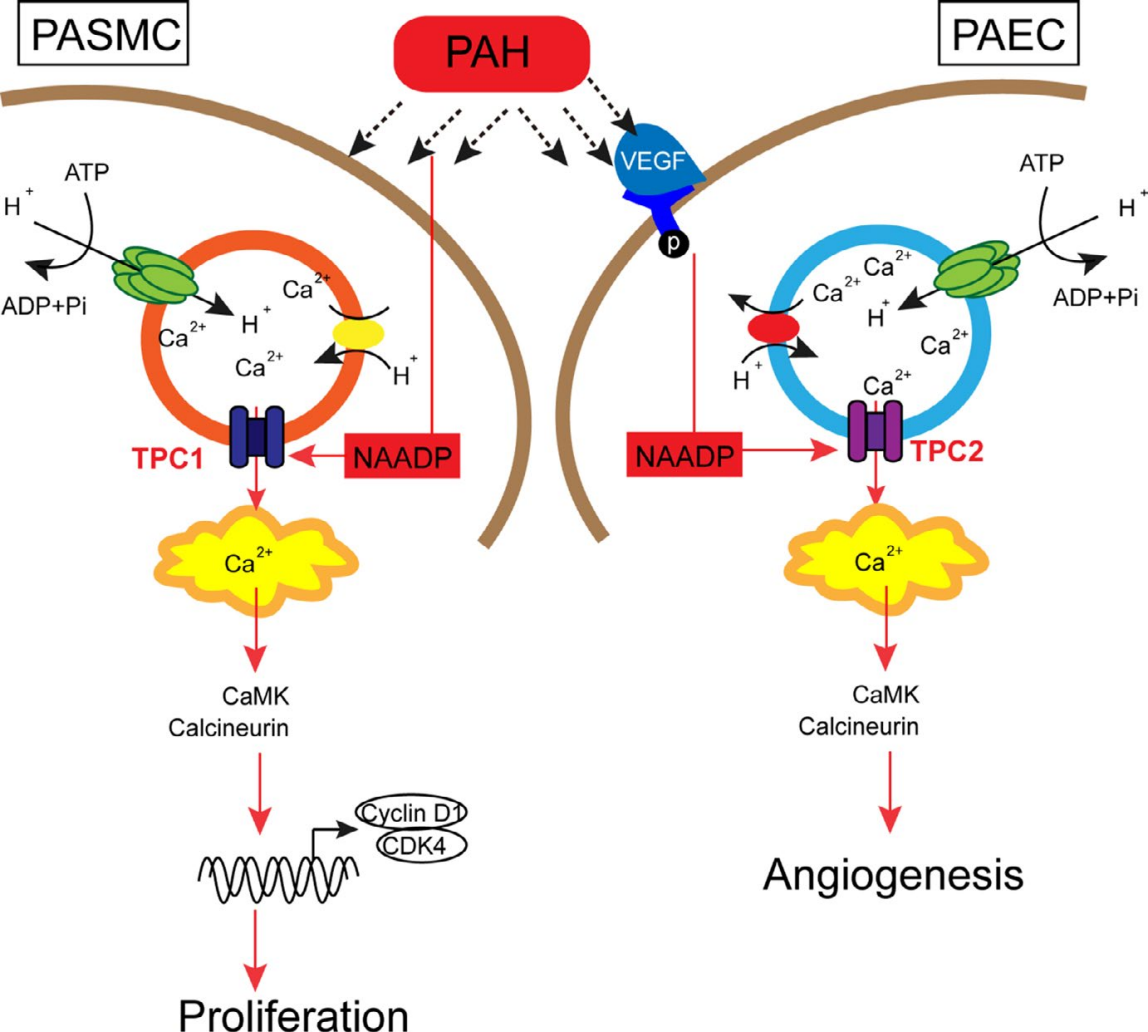
Schematic diagram exhibiting NAADP‐induced Ca release is mediated by TPC1/2 in hypoxia‐induced pulmonary arterial hypertension

Herein, it is confirmed that TPC1/2 plays essential roles in NAADP‐mediated calcium release within PAH rat models. Within PASMCs, TPC1 plays a dominant role by regulating NAADP‐mediated calcium release in PASMCs and PASMC proliferation; within PAECs, TPC2 plays a dominant role through the regulation of NAADP‐mediated calcium release within PAECs and PAEC angiogenesis (Figure 8).

## 4 DISCUSSION

Pulmonary arterial hypertension induced by hypoxia is a life‐threatening progressive disorder characterized by abnormal blood pressure levels in pulmonary arteries. Thorough studies provided evidence that excessive proliferation of PASMC and the angiogenesis of PAECs could be correlated with the enhanced activation of Ca^2+^ signalling transduction.^37^, ^38^, ^39^ The hypoxia‐induced rat PAH model was established to further investigate the underlying mechanisms, then evaluated by the arterial wall thickness, mean pulmonary artery pressure and the RV/LS + S weight ratio.

TPC gene family encodes a newly identified protein involved in the endolysosome‐targeted calcium release gated by NAADP, a Ca^2+^ mobilizer stored within cells.^40^, ^41^ It has been recently reported that the two‐pore channels (TPCs), including TPC1 and TPC2, are the NAADP‐activated calcium release channels. As reported by Castonguay et al, whereas TPC1 shows an apparent broad pattern of colocalization with markers for recycling endosomes, early and late endosomes, and lysosomes,^42^ TPC2 predominantly colocalizes with markers for late endosomes and lysosomes.^43^, ^44^, ^45^, ^46^ As confirmed by functional investigations, TPC1/2 overexpression promotes the NAADP‐mediated calcium response ^43^, ^44^; TPC reconstitution within lipid bilayers depicts the activity of NAADP‐mediated channels ^47^, ^48^; and endogenous TPC silence or tpcn2 gene deletion can potentially attenuate NAADP‐mediated responses within intact native cells.^48^, ^49^, ^50^ Our previous study demonstrated that both TPC1 and TPC2 express in PASMCs, with TPC1 being the dominant subtype ^27^; however, their respective roles and the detailed mechanisms have not yet been entirely determined as of yet. Herein, NAADP concentration and TPC1/2 mRNA and protein levels within pulmonary tissues derived from hypoxia‐activated PAH rat model were evaluated; consistent with our previous study, NAADP content and TPC1/2 mRNA and protein levels were significantly increased within PAH tissue samples than that from the normal group, indicating the potential roles of TPC1/2. Through lentivirus infection containing shRNA of TPC1/2, TPC1/2 knockdown was achieved, and the PAH indicators were checked to evaluate the functions of TPCs. After TPC knockdown, PAH was alleviated, manifested as improved arterial wall thickness, partially declined the mean pulmonary artery pressure and RV/LS + S weight ratio, under hypoxic but not under normoxic condition. How do TPCs exert their functions in the regulation of hypoxia‐induced PAH in rat models? The underlying mechanisms have been further investigated.

Given the essential roles of Ca^2+^ release in PAH, whether TPC knockdown could act on [Ca^2+^]*_i_* in PASMCs was initially investigated. Upon NAADP‐AM stimulation, [Ca^2+^]*_i_* in PASMCs immediately presented a marked increase; after TPC1/2 knockdown by Lsh‐TPC1/2 infection, [Ca^2+^]*_i_* was reduced; however, TPC1/2 knockdown exhibited different inhibitory efficiency. Compared to the Lsh‐NC group, TPC1 knockdown sharply down‐regulated [Ca^2+^]*_i_*, whereas TPC2 knockdown only caused a slight down‐regulation on [Ca^2+^]*_i_*, suggesting the dominant effect of TPC1 on regulating [Ca^2+^]*_i_* within PASMCs. To further confirm our hypothesis, the mRNA and protein level changes of Ca^2+^ signalling‐related factors were subsequently monitored CaMKII and calcineurin in PASMCs. Consistent with [Ca^2+^]*_i_* determination, NAADP‐AM markedly increased CaMKII and calcineurin mRNA and protein expression within PASMCs. After TPCs knockdown, different results were yielded. TPC2 knockdown caused a slight reduction on CaMKII and calcineurin mRNA and proteins, whereas TPC1 knockdown or TPC blocker sharply suppressed CaMKII and calcineurin mRNA and proteins. As mentioned earlier, excessive proliferation of PASMC and PAEC could potentially trigger vascular remodelling, which alters the pulmonary artery structures along with its biochemical and functional phenotypes.^9^ The function of TPC1 was equally evaluated in the regulation of PASMC proliferation. Interestingly, single TPC1 knockdown only moderately but not significantly suppressed PASMC proliferation, whereas it significantly reversed NAADP‐AM‐induced PASMC proliferation, thus further indicating that TPC1 exerts a dominant effect on regulating [Ca^2+^]*_i_* within PASMCs and PASMC proliferation.

In addition to TPC1, the calcium release from acidic compartments via TPC2 activation evoked by NAADP could be observed in different cell types such as megakaryoblastic cell lines, pulmonary arterial myocytes, gastric smooth muscle cells and mouse embryonic stem cells.^27^, ^45^, ^51^, ^52^ In cells, it modulated store‐operated Ca^2+^ entry and linked to lysosomal functions such as alkalinizing lysosomal pH, endolysosomal morphology and cell pigmentation.^53^, ^54^, ^55^ TPC2 was also found in connection to autophagic signallings, such as skeletal muscle or cultured astrocytes.^56^, ^57^, ^58^ As revealed by the other reports, a VEGFR2/NAADP/TPC2/Ca^2+^ signalling plays a crucial role in VEGF‐mediated proliferation and angiogenesis in vitro and in vivo.^28^ It is supposed that TPC2 might also have a role in hypoxic exposure‐induced [Ca^2+^]*_i_* and PAEC angiogenesis. However, its potential role in PAH has not yet been determined as of yet. The aforementioned assays in PAECs were performed to evaluate TPC1/2 functions. Consistent with in PASMCs, NAADP‐AM also induced [Ca^2+^]*_i_* and Ca^2+^‐related CaMKII and calcineurin expression in PAECs. As expected, TPC2 knockdown or TPC blocker sharply reversed NAADP‐induced [Ca^2+^]*_i_*, as well as NAADP‐AM‐induced CaMKII and calcineurin expression, whereas TPC1 knockdown only resulted in slight changes of the indicated indexes in PAECs, suggesting the dominant effect of TPC2 on regulating [Ca^2+^]*_i_* within PAECs. Also, the effect of TPC2 on regulating PAEC angiogenesis was monitored. As expected, TPC2 knockdown significantly inhibited VEGF‐induced angiogenesis of PAEC, further confirming the dominant role of TPC2 in PAECs.

TPC1 has an essential role in controlling excessive PASMC proliferation through regulation of [Ca^2+^] *_i_* in PASMCs during pulmonary artery hypertension. Meanwhile, TPC2 exerts a crucial effect on controlling excessive PAEC angiogenesis by regulating [Ca^2+^]*_i_* in PAECs. A theoretical basis of TPC1/2 serving as potential targets for new gene treatments of chronic hypoxic‐related lung diseases, including PAH, was provided. However, as for NAADP‐induced TPC activation, it is possible that NAADP might target a secondary protein which either closely binds to TPCs or translocates to TPCs after NAADP binding to form part of the TPC complex other than direct binding to TPCs,^59^, ^60^, ^61^ Recently, JPT2 has been identified as NAADP‐binding protein which acts as TPC accessory protein to participate the endogenous NAADP‐evoked Ca signalling.^61^ However, the specific function of NAADP‐binding proteins during PAH requires further validation in future studies.

## CONSENT FOR PUBLICATION

Not applicable.

## CONFLICT OF INTEREST

The authors declare that they have no conflict of interests.

## AUTHOR CONTRIBUTION

**Wen Hu:** Conceptualization (equal); Data curation (equal); Methodology (equal); Writing‐original draft (equal). **Fei Zhao:** Resources (equal); Software (equal); Visualization (equal). **Ling Chen:** Supervision (equal); Validation (equal). **Jiamin Ni:** Resources (equal); Software (equal); Validation (equal). **Yongliang Jiang:** Conceptualization (equal); Funding acquisition (equal); Project administration (equal); Writing‐review & editing (equal).

## ETHICAL APPROVAL

The study was conducted in accordance with the Declaration of Helsinki, and the protocol was approved by the Ethics Committee of the Hunan Provincial People's Hospital. Prior consent was obtained from each patient enrolled.

## Supporting information

Fig S1‐5

## Data Availability

The authors confirm that the data supporting the findings of this study are available within the article and its supplementary materials.
