# Supplementary material for: NAADP‐induced intracellular calcium ion is mediated by the TPCs (two‐pore channels) in hypoxia‐induced pulmonary arterial hypertension
Source: J Cell Mol Med. 2021 Jul 15;25(15):7485–99. doi: 10.1111/jcmm.16783 (PMC8335677; doi:10.1111/jcmm.16783)
Supplement: Supplementary file 1 — Fig S1‐5 [file JCMM-25-7485-s001.docx]

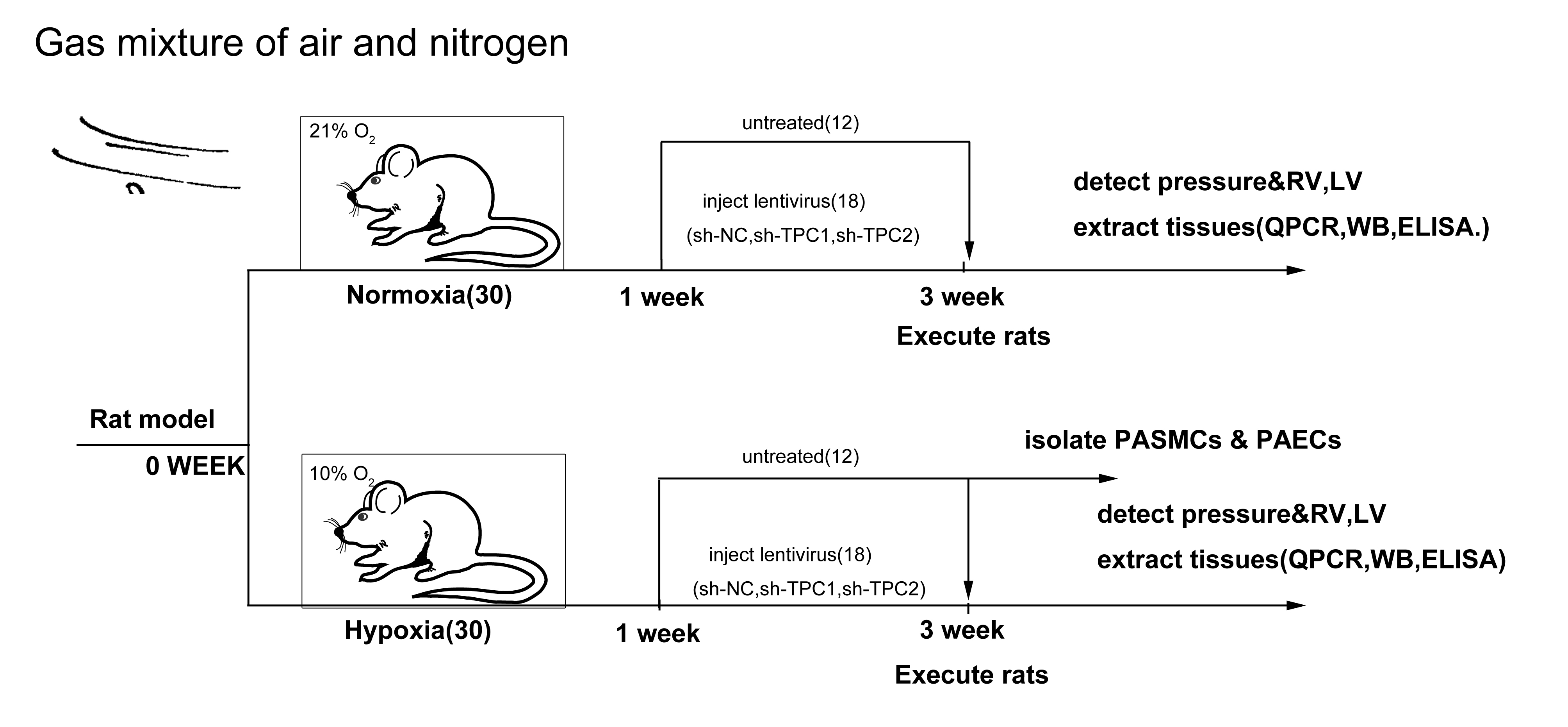


**Figure S1 The flow chart of animal model building and further experiments to assess the effect of TPC1/2 on PAH, PASMCs, and PAECs.**


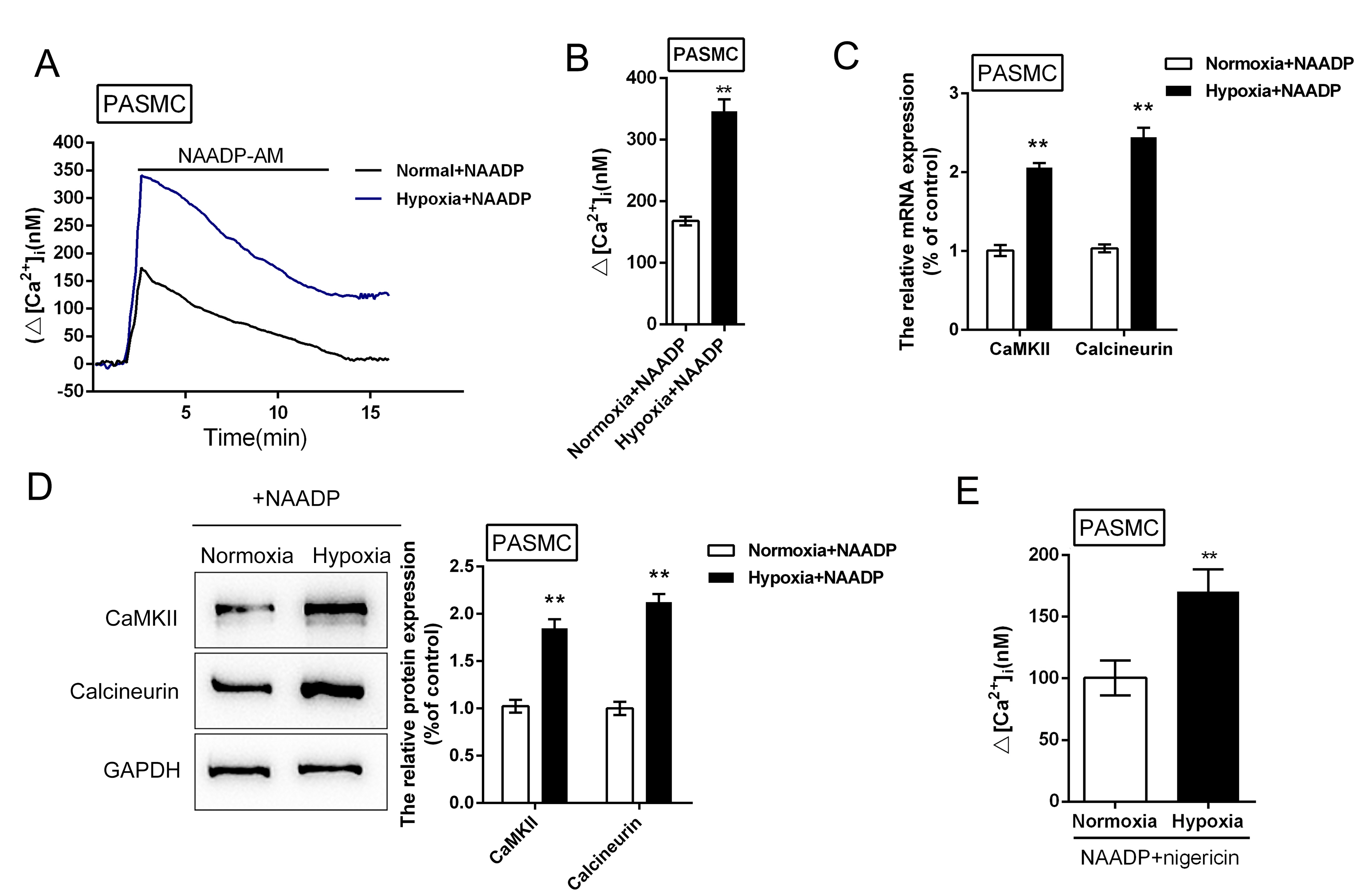


**Fig.S2 NAADP-AM-induced [Ca^2+^]*_i_* and Ca^2+^ signal markers were determined in PASMCs in response to hypoxia.** (A-B) [Ca^2+^]*_i_* in PASMCs with the NAADP-AM stimulation was determined. (C-D) The mRNA and protein levels of CaMKII and Calcineurin. (E) Under nigericin treatment, NAADP-AM-induced [Ca2+]*i* in PASMCs were determined. P<0.01


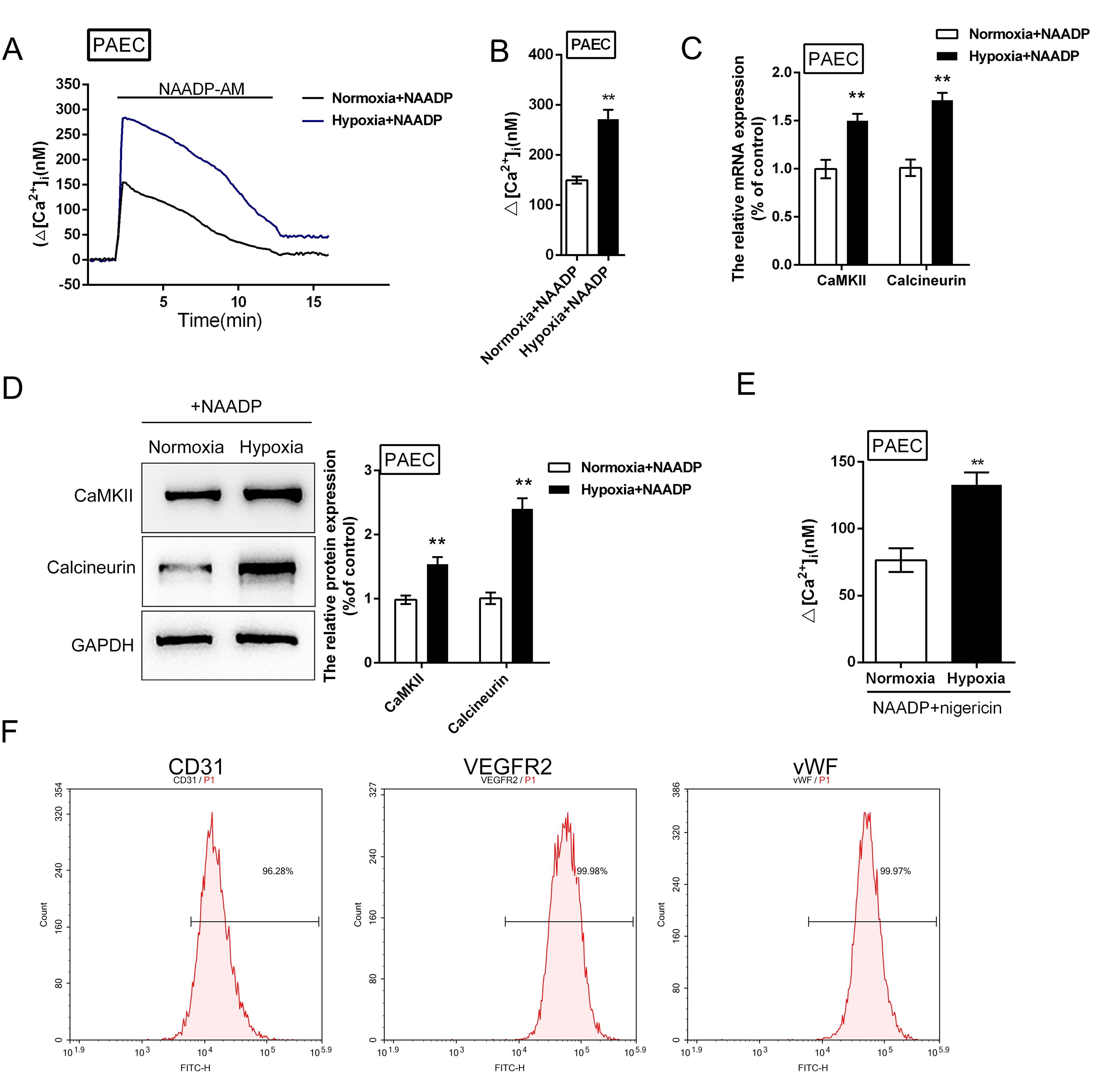


**Fig.S3 NAADP-AM-induced [Ca^2+^]*_i_* and Ca^2+^ signal markers were determined in PAECs in response to hypoxia.** (A-B) [Ca2+]*i* in PAECs with the NAADP-AM stimulation was determined. (C-D) The mRNA and protein levels of CaMKII and Calcineurin. (E) Under nigericin treatment, NAADP-AM-induced [Ca2+]*i* in PAECs were determined. (F) the identification of PAECs by determining the expression of CD31, VEGFR2 and vWF using flowcytometry. P<0.01


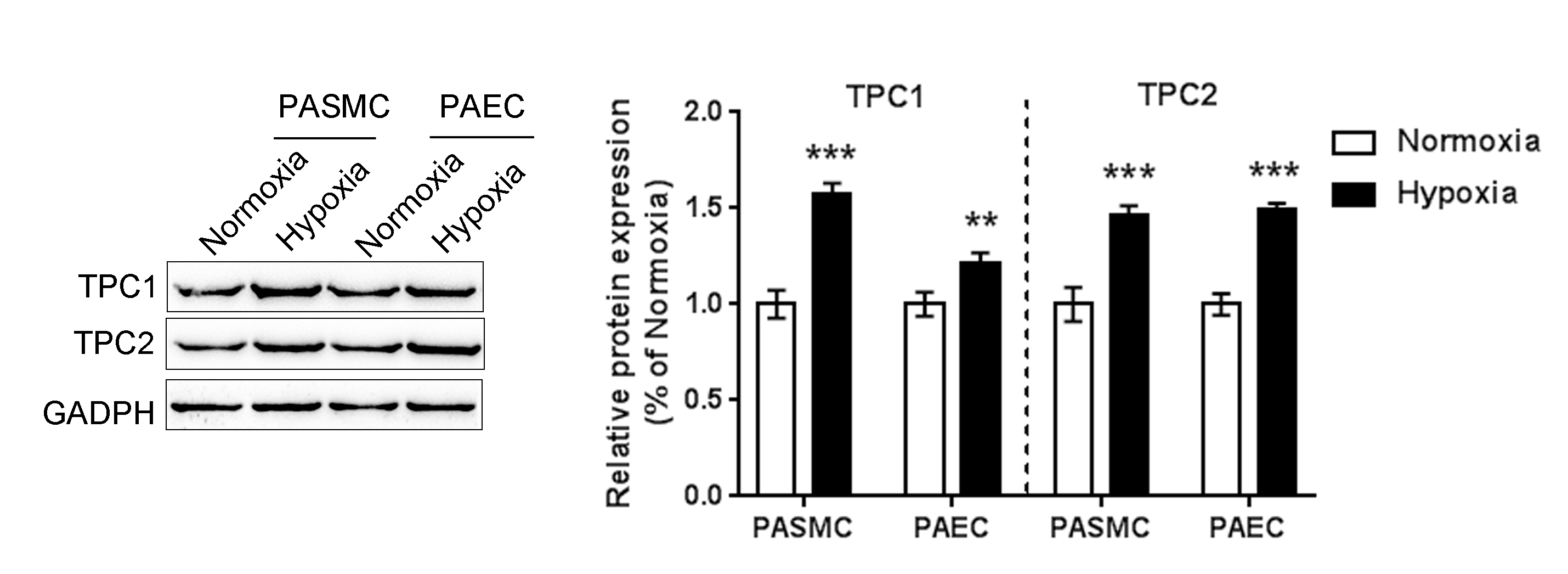


**Fig.S4 The protein levels of TPC1/2 in response to hypoxia in PASMCs and PEACs examined by Immunoblotting**


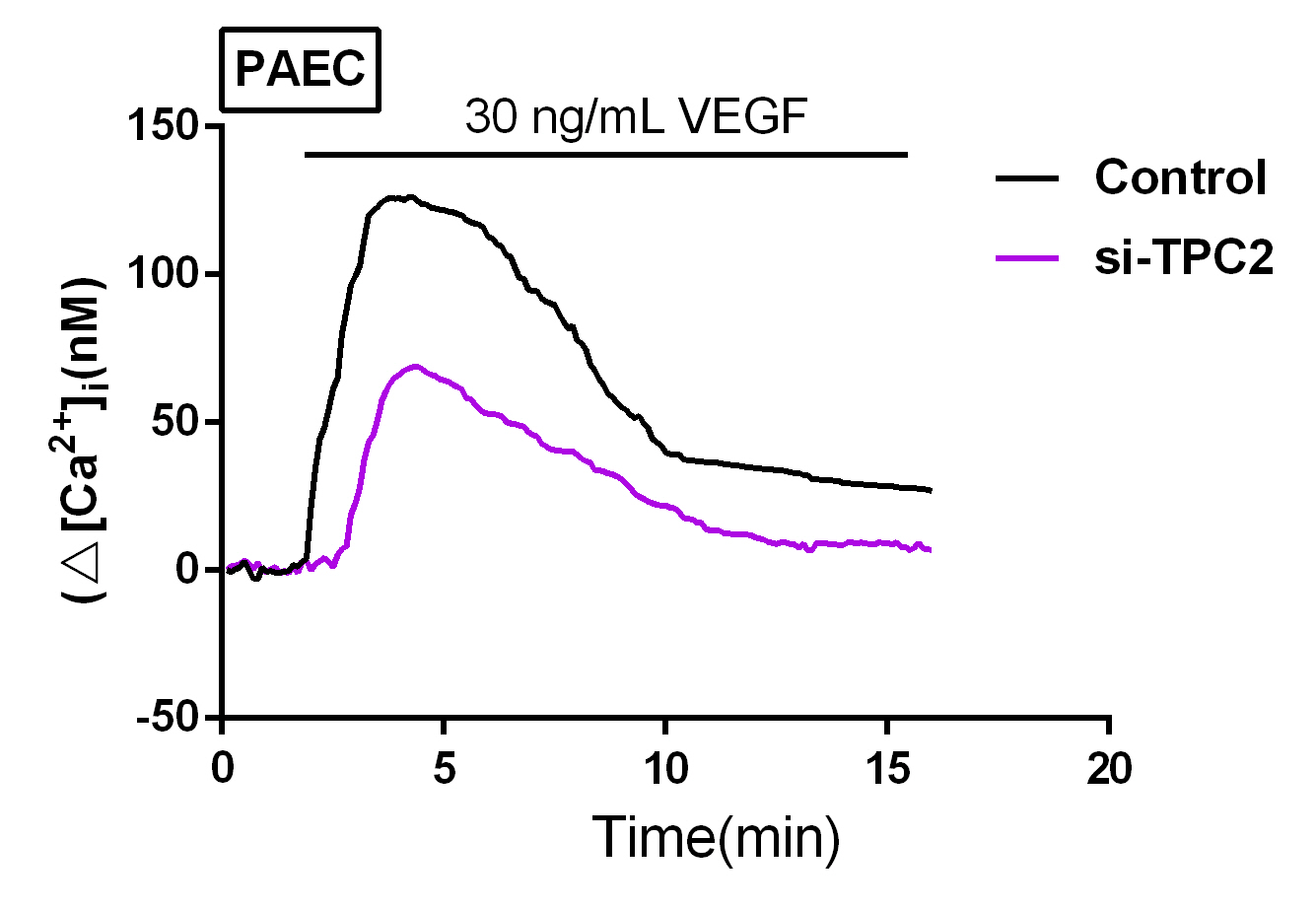


**Fig.S5 VEGF-induced [Ca^2+^]*_i_* in response to TPC2 silence**
